# Retinoid Acid Specifies Neuronal Identity through Graded Expression of Ascl1

**DOI:** 10.1016/j.cub.2013.01.046

**Published:** 2013-03-04

**Authors:** John Jacob, Jennifer Kong, Steven Moore, Christopher Milton, Noriaki Sasai, Rosa Gonzalez-Quevedo, Javier Terriente, Itaru Imayoshi, Ryoichiro Kageyama, David G. Wilkinson, Bennett G. Novitch, James Briscoe

**Affiliations:** 1 Division of Developmental Biology, MRC National Institute for Medical Research, The Ridgeway, Mill Hill, London NW7 1AA, UK; 2 Division of Developmental Neurobiology, MRC National Institute for Medical Research, The Ridgeway, Mill Hill, London NW7 1AA, UK; 3 National Hospital for Neurology and Neurosurgery, Queen Square, London WC1N 3BG, UK; 4 Department of Neurobiology, David Geffen School of Medicine at UCLA, 610 Charles E. Young Drive E, Los Angeles, CA 90095, USA; 5 Broad Center of Regenerative Medicine and Stem Cell Research, David Geffen School of Medicine at UCLA, 610 Charles E. Young Drive E, Los Angeles, CA 90095, USA; 6 Laboratory of Growth Regulation, Institute for Virus Research, Kyoto University, Shogoin-Kawahara, Sakyo-ku, Kyoto 606- 8507, Japan

## Abstract

Cell diversity and organization in the neural tube depend on the integration of extrinsic signals acting along orthogonal axes. These are believed to specify distinct cellular identities by triggering all-or-none changes in expression of combinations of transcription factors [1]. Under the influence of a common dorsoventral signal, sonic hedgehog, and distinct anterior-posterior (A-P) inductive signals [2, 3], two topographically related progenitor pools that share a common transcriptional code produce serotonergic and V3 neurons in the hindbrain and spinal cord, respectively [4–7]. These neurons have different physiological properties, functions, and connectivity [8, 9]. Serotonergic involvement in neuropsychiatric diseases has prompted greater characterization of their postmitotic repertoire of fate determinants, which include Gata2, Lmx1b, and Pet1 [10], whereas V3 neurons express Sim1 [4]. How distinct serotonergic and V3 neuronal identities emerge from progenitors that share a common transcriptional code is not understood. Here, we show that changes in retinoid activity in these two progenitor pools determine their fates. Retinoids, via Notch signaling, control the expression level in progenitors of the transcription factor Ascl1, which selects serotonergic and V3 neuronal identities in a dose-dependent manner. Therefore, quantitative differences in the expression of a single component of a transcriptional code can select distinct cell fates.

## Highlights

► Graded Ascl1 expression in the neural tube specifies distinct neuronal subtypes ► Retinoid activity and Ascl1 expression are inversely correlated ► Binary differences in retinoid signaling regulate graded Ascl1 expression via Notch ► Dose-dependent effects of Ascl1 explains regional segregation of neuronal subtypes

## Results

Through the inductive effects of signaling molecules such as sonic hedgehog [4] and fibroblast growth factors [3], hindbrain serotonergic (5HT) progenitors acquire a Foxa2^+^Nkx2.2^+^Ascl1^+^ transcriptional code. Topographically related V3 neural progenitors in the spinal cord express the same three factors (see Figure S1A available online). All three factors have been shown to be necessary for the generation of 5HT neurons, and Nkx2.2 and Ascl1 are critical for V3 neurogenesis [4, 5, 11, 12]. Consistent with its expression in spinal cord p3 progenitors, Foxa2 is also required for the generation of V3 neurons (Figures S1B, S1D, and S1E) [13]. This raises the question of how hindbrain and spinal p3 progenitors, designated p3[5HT] and p3[V3], respectively, generate different cell types.

### Retinoids Regulate p3 Progenitor Identity

**Figure 1:**
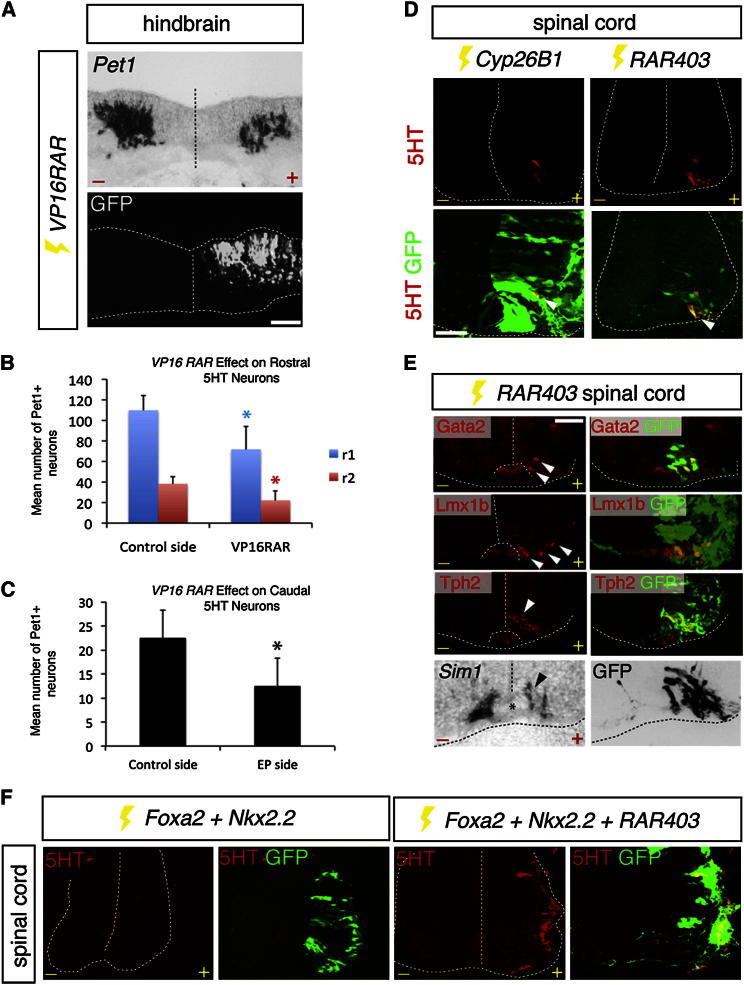
Retinoid Signaling Determines p3 Progenitor Fate (A) Upregulation of RA signaling in hindbrain p3[5HT] progenitors by misexpression of *VP16RAR-IRES GFP* reduces *Pet1* expression. Scale bar represents 25 μm. (B and C) Effect of *VP16RAR* misexpression on *Pet1*^*+*^ neurons in the rostral (B) or caudal (C) hindbrain at E5. ^∗^p = 0.025 (B, blue bar), ^∗^p = 0.026 (B, red bar), ^∗^p = 8.11 × 10^−6^ (C, black bar) (unpaired Student’s t tests). Error bars represent SD in all graphs. r, rhombomere. (D) Forced expression of *Cyp26B1* or *RAR403* in p3[V3] generates ectopic 5HT neurons (arrowheads) by E5. Scale bar represents 14 μm. (E) Ectopic spinal 5HT neurons express the indicated markers (arrowheads), and *Sim1* expression is reduced. Asterisk marks the floor plate. Scale bar represents 16 μm. (F) *Nkx2.2* and *Foxa2* misexpression only induce ectopic spinal 5HT neurons when RA signaling is blocked by *RAR403*. –, control side; +, electroporated side. See also Figure S2.

We analyzed β-gal expression in the hindbrain and spinal cord of *RARE-LacZ* transgenic reporter mice [14]. The hindbrain has a segmental organization and is composed of eight compartments termed rhombomeres [15]. β-gal immunostaining revealed low or absent RA signaling in p3[5HT] progenitors at multiple rhombomeric levels, but high levels of activity in p3[V3] cells (Figure S2A). This is consistent with other assays of retinoid signaling in amniotes (for example, see [16, 17]) and suggested that retinoid signaling could be involved in p3 progenitor fate determination. To test this, we took advantage of a constitutively active retinoid receptor α derivative, *VP16RAR*, which has previously been shown to activate retinoic acid receptor target genes independently of RA [18]. Forced expression of *VP16RAR* by in ovo electroporation reduced 5HT neuronal differentiation by 40%–50% throughout the A-P extent of the hindbrain (Figures 1A–1C). Concomitantly, occasional ectopic V3 neurons, marked by expression of the postmitotically expressed transcription factor *Sim1*, were produced in the caudal hindbrain (data not shown) [4]. Conversely, blockade of retinoid signaling in p3[V3] progenitors, either by misexpression of *Cyp26B1*, a member of the cytochrome P450 class of enzymes that degrades retinoic acid, or *RAR403*, a dominant negative version of the human RA receptor α [19], resulted in ectopic 5HT neuronal generation (Figure 1D). These neurons expressed the postmitotic serotonergic determinants Gata2 [20] and Lmx1b [21, 22], but not Pet1 [23], and they also expressed the terminal differentiation marker Tph2 [24] (Figure 1E). Therefore, ectopic spinal 5HT neurons share the same differentiation pathway as hindbrain 5HT neurons, of which approximately one-third do not depend on Pet1 for their generation [25, 26].

The ventral restriction of ectopic spinal 5HT neurons suggested their derivation from presumptive p3[V3] progenitors, and blockade of Nkx2.2 or Foxa2 activity using a dominant interfering derivative of Nkx2.2 (*Nkx2.2-VP16*) [27] or Foxa2 (*Fkh*^*A2*^*-EnR*) [5], respectively, prevented *RAR403* induction of ectopic 5HT neurons (Figure S2B). Consistent with these data, forced expression of *Foxa2*, *Nkx2.2*, and *RAR403* in the dorsal neural tube led to extensive ectopic generation of 5HT neurons, whereas the omission of *RAR403* led to a failure to induce ectopic 5HT neurons (Figure 1F). This result also suggested that the small number of ectopic 5HT neurons generated by p3[V3] in ovo electroporation with *RAR403* is likely to be a consequence of the restricted overlap of endogenous Foxa2 and Nkx2.2 expression and mosaic misexpression of *RAR403*. Furthermore, as quantified below, at brachial level the p3 domain normally only generates relatively small numbers of V3 neurons, suggesting that low neurogenic potential might further limit ectopic 5HT neurogenesis.

V3 neuronal differentiation was suppressed by 60% upon *RAR403* misexpression (V3 neurons in control = 10.7 [mean] ± 4.1 [SD]; V3 neurons following *RAR403* misexpression = 4.7 ± 2.2; p = 7.18 × 10^−9^, unpaired Student’s t test) (Figure 1E). This was not due to cell death or to a fate transformation to other ventral neuronal subtypes (Figure S2C). Furthermore, ectopic visceral motor (VM) neurons that precede the generation of 5HT neurons in the hindbrain from p3 progenitors [6] (Figure S1A) were not detected (Figure S2C). Altogether, these data indicate that high-level, versus low-level or absent, retinoid signaling in p3[V3] and p3[5HT], respectively, determines the corresponding neuronal identities.

### Retinoids Regulate Ascl1 Expression in p3 Progenitors via the Notch Pathway

**Figure 2:**
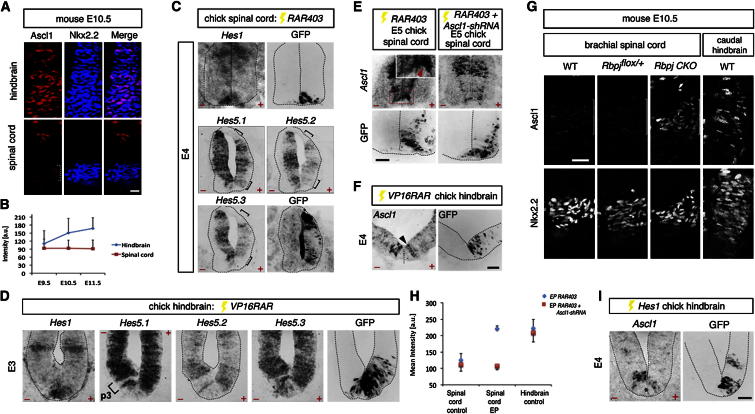
Ascl1 Expression Level in p3 Progenitors Is Negatively Correlated with Notch Activity, which in Turn Is Regulated by Retinoids (A and B) Ascl1 expression in mouse Nkx2.2^+^ p3 progenitors (A), with quantification (mean ± SD) shown in (B). Scale bar represents 9 μm. a.u., arbitrary units. n = 3 sections from each of three embryos per developmental stage. (C) Spinal *RAR403* misexpression, marked by anti-GFP immunofluorescence, downregulates *Hes* gene expression (bracketed). (D) Misexpression of *VP16RAR* upregulates hindbrain p3 *Hes* gene expression. (E) Misexpression of *RAR403* in p3[V3] progenitors upregulates *Ascl1* expression (arrowhead, inset) at E5, and this is prevented by *cAscl1-shRNA*. Inset shows a high-power image of the boxed region. Scale bar represents 18 μm. (F) *VP16RAR* misexpression attenuates *Ascl1* expression (arrowhead) in p3[5HT] progenitors. Scale bar represents 18 μm. (G) Increased Ascl1 expression in brachial Nkx2.2^+^ p3[V3] cells (white brackets) of *RbpjCKO* mouse embryos is similar to Ascl1 expression in WT p3[5HT] cells. Scale bar represents 14 μm. (H) Quantification of *Ascl1* expression in presumptive p3[V3] cells misexpressing *RAR403* or *RAR403* and *cAscl1-shRNA*. (I) Misexpression of *Hes1-IRES GFP* in caudal hindbrain p3[5HT] reduces *Ascl1* expression. Scale bar represents 18 μm. See also Figure S1.

The profile of RA signaling in the ventral neural tube correlates with the longitudinal expression of Ascl1 in p3 progenitors (Figures S1C and S2A). The significance of this observation lies in the critical subtype-specification function of *Ascl1* in p3[5HT] and p3[V3] progenitors, which is separable from its neurogenic properties [11, 12]. In the p3[5HT] domain where RA signaling is weak or absent, the expression level of chick *Ascl1* appeared markedly higher than in the p3[V3] domain where RA signaling is strong (Figure S1C). This observation prompted closer scrutiny of the expression of Ascl1 in p3 progenitors in the spinal cord and hindbrain (Figures 2A and 2B). Indeed, the mean level of Ascl1 expression was consistently greater in p3[5HT] than in p3[V3] progenitors between E9.5 and E11.5. Moreover, Ascl1 expression level was enhanced in hindbrain p3 progenitors after E9.5, corresponding to the time 5HT neurogenesis commences [5].

The Notch pathway is known to inhibit proneural gene expression, and, consistent with this regulatory relationship, in chick p3 progenitors the *Hes* family of Notch target genes showed reciprocal patterns of expression to *Ascl1* (Figures S2D and S2E). Moreover, the Notch pathway lies downstream of retinoid signaling. Misexpression of *RAR403* in the p3[V3] domain led to the downregulation of the Notch pathway by E4 (Figure 2C). Conversely, forced expression of *VP16RAR* in the hindbrain p3[5HT] domain upregulated Notch activity (Figure 2D). Retinoid regulation of the Notch pathway suggested Notch signaling might serve to link the A-P pattern of retinoid activity and Ascl1 expression in p3 progenitors.

To test whether the expression level of Ascl1 in p3[5HT] and p3[V3] is regulated by retinoid signaling, we analyzed *Ascl1* expression in chick p3 progenitors following cell-autonomous blockade or activation of RA signaling. *RAR403* markedly upregulated *Ascl1* expression in the p3[V3] domain by E5 24 hr after the downregulation of the Notch pathway (control p3[V3] expression = 126 [mean] ± 19.3 [SD] [arbitrary units]; *RAR403* p3[V3] expression = 221.6 ± 7.8; control p3[5HT] expression = 223.1 ± 26.3). (Figures 2E and 2H; Figure S2F). Moreover, the upregulation of *Ascl1* could be prevented by the comisexpression of a short hairpin RNA against chick *Ascl1* (*cAscl1-shRNA*) (control p3[V3] expression = 110.9 [mean] ± 19.4 [SD]; *RAR403 + cAscl1-shRNA* p3[V3] expression = 107.1 ± 11.2; control p3[5HT] expression = 208.4 ± 27.5) (Figures 2E and 2H). By contrast, misexpression of *VP16RAR* in p3[5HT] progenitors downregulated *Ascl1* by E4 1 day after the upregulation of the Notch pathway in these progenitors (control p3[5HT] expression = 193.1 [mean] ± 27.1 [SD]; *VP16RAR* p3[5HT] expression = 94.4 ± 16.9; control p3[V3] expression = 105.1 ± 16.9) (Figure 2F). In neither condition were there detectable changes in expression of the other proneural genes, *Neurog1* and *Neurog2*, or of other ventral progenitor markers or sonic hedgehog. Moreover, there was no increase in cell death (Figures S2F–S2H).

To determine whether Notch signaling regulates Ascl1 expression in p3 progenitors, we conditionally deleted recombination signal binding protein for immunoglobulin kappa j region (*Rbpj*), an obligate component of the Notch signaling pathway [28], using *Olig2-cre* [29]. At E10.5, mice lacking *Rbpj* (*RbpjCKO*) expressed higher levels of Ascl1 in the spinal cord p3 domain, compared to either mice that have only one conditional allele or mice that lacked *Cre* (WT p3[V3] expression: 50.5 [mean Ascl1 expression] ± 28.0 [SD]; *Rbpj*^*flox/+*^ p3[V3] expression: 49.3 ± 29.0; *RbpjCKO* p3[V3] expression: 78.1 ± 50.4; WT p3[5HT] expression: 90.3 ± 56.8) (Figure 2G). Conversely, misexpression of *Hes1*, an effector of Notch signaling, in the caudal hindbrain of the chick lowered the expression of *Ascl1* in p3[5HT] progenitors to levels comparable to the expression of *Ascl1* in spinal cord p3 progenitors (control p3[5HT] = 221.9 [mean] ± 20.9 [SD]; *Hes1* electroporation p3[5HT] = 123.4 ± 8.7; control p3[V3] = 119.8 ± 22.1) (Figure 2I). The expression of Foxa2 and Nkx2.2 were unaffected (Figure S2I). Together, these gain- and loss-of-function data suggested that differential expression of Ascl1 in the two p3 progenitor pools is determined by retinoid signaling acting via the Notch pathway. Furthermore, our findings raised the possibility that differences in the expression level of Ascl1 might determine p3 progenitor fate.

### Ascl1 Expression Level Discriminates p3[V3] from p3[5HT] Progenitors

**Figure 3:**
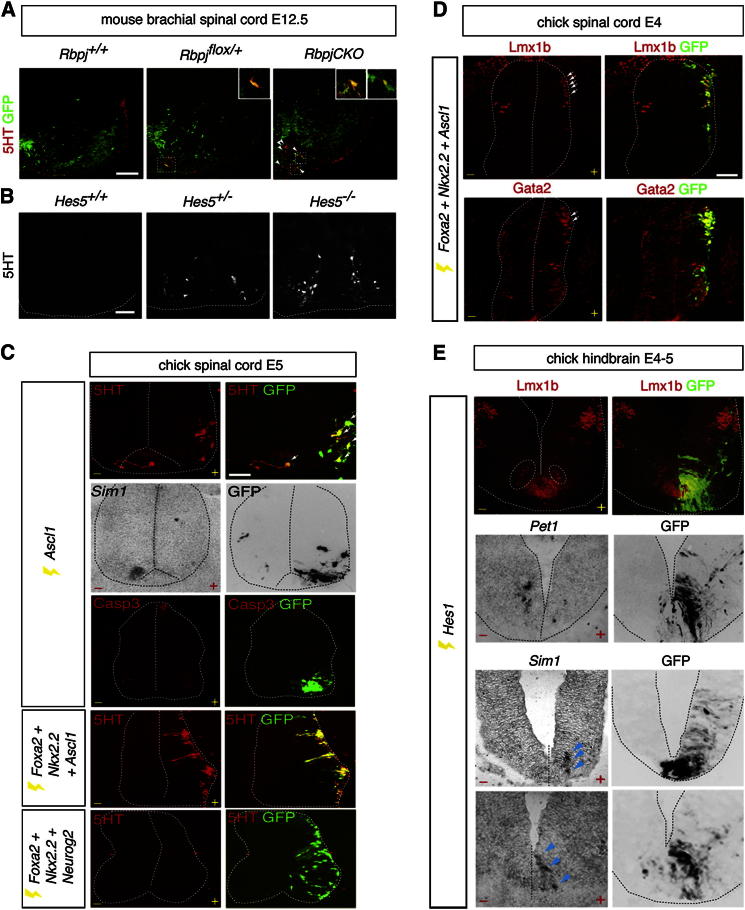
Altering Ascl1 Expression Level in p3 Progenitors Produces Corresponding Changes in p3 Progenitor Identity (A and B) Ectopic 5HT^+^GFP^+^ neurons (yellow cells, arrowheads) in the cervical cord of *Rbpj* mutants (A) and 5HT^+^ cells in the brachial cord of *Hes5* mutants (B). Insets show high-power images of 5HT^+^GFP^+^ neurons in boxed regions. Scale bar represents 25 μm in (A) and 13 μm in (B). (C) Forced *Ascl1* expression in cervicobrachial p3[V3] cells induces ectopic 5HT neurons (arrows) and reduces V3 differentiation. Cell death, assayed by activated Caspase3 expression, is not detected in the ventral spinal cord. *Ascl1*, but not *Neurog2*, substitutes for *RAR403* in combined electroporation with *Foxa2* and *Nkx2.2* to induce ectopic spinal 5HT neurons. Scale bar represents 13 μm. (D) Ectopic Lmx1b and Gata2 expression (arrows) upon combined *Foxa2*, *Nkx2.2*, and *Ascl1* electroporation at brachial level. Scale bar represents 14 μm. (E) *Hes1-IRES GFP* misexpression in caudal p3[5HT] cells reduces 5HT neuronal differentiation marked by Lmx1b (circled) and *Pet1* and induces ectopic V3 neurons (arrowheads).

We exploited the regulatory relationship between Notch signaling and proneural gene expression to test the effect of boosting Ascl1 expression in the spinal cord on p3 progenitor fate. Strikingly, although 5HT neurons are normally never found in this region of the neural tube, ectopic spinal 5HT^+^GFP^+^ neuronal cell bodies were observed in the cervical cords of nine out of nine *Rbpj* conditional null mice and two out of five Cre/+; *Rbpj*^*flox/+*^ mice (Figure 3A). Moreover, analysis of embryos lacking *Hairy* and *Enhancer-of-Split* (*Hes*) family member *Hes5,* which is a target of Notch signaling broadly expressed in the spinal cord [30] (Figure 3B), revealed ectopic 5HT neurons in the ventral spinal cord at forelimb levels in three out of four *Hes5*^*+/−*^ embryos and four out of four *Hes5* null embryos. These neurons were more abundant than in *Rbpj* mutants: the later deletion of *Rbpj* in neural cells by *Olig2-cre* (∼E9.5) might explain the smaller number of 5HT neurons.

To further explore the role of Ascl1 expression level in regulating p3 progenitor fate, we first increased *Ascl1* levels in the p3[V3] domain by direct overexpression in ovo (Figure 3C). Boosting the level of Ascl1 in the p3[V3] domain was sufficient to generate ectopic 5HT neurons cell autonomously and suppress V3 neuronal differentiation (the number of *Sim1*^+^ cells on the electroporated side was 1.59 [mean] ± 1.6 [SD], and on the control side there were 8.12 ± 2.3, p = 2.80 × 10^−10^; n = 18 sections from five embryos), indicating that upregulation of Ascl1 reprograms the p3[V3] domain to a p3[5HT] identity. Moreover, there was no evidence that cell death, as revealed by activated Caspase3 immunostaining, could account for the reduced number of V3 neurons (Figure 3C). Next, we misexpressed the three p3-defining transcription factors, *Nkx2.2*, *Foxa2*, and *Ascl1*, in the dorsal spinal cord. The widespread and cell-autonomous generation of ectopic 5HT neurons that resulted shows that *Ascl1* can substitute for *RAR403* and supports the view that high-level Ascl1 expression is sufficient to select a p3[5HT] identity over a p3[V3] identity (Figure 3C). Ectopic 5HT neurons expressed postmitotic markers of hindbrain 5HT neurons Lmx1b and Gata2, but not *Pet1* (Figure 3D and data not shown), suggesting that the differentiation of these neurons largely recapitulates the serotonergic differentiation pathway. Importantly, neither overexpression of *Foxa2* and *Nkx2.2* alone nor replacement of *Ascl1* by another bHLH gene, *Neurog2*, led to ectopic 5HT neuronal generation (Figures 1F and 3C). Therefore, increasing the expression of *Ascl1* converts p3[V3] to a p3[5HT] identity.

We then asked whether reducing the expression of *Ascl1* in presumptive p3[5HT] progenitors would cause a reciprocal switch to a p3[V3] identity. Following *Hes1* in ovo electroporation into presumptive p3[5HT] progenitors, significant reductions in 5HT neuronal differentiation were observed, using Lmx1b and *Pet1* as markers (control side 5HT neurons = 8.2 [mean] ± 2.8 [SD]; *Hes1*-electroporated side = 2.5 ± 2.1, p = 8.2 × 10^−8^), and small numbers of *Sim1*-expressing ectopic V3 neurons were consistently detected in the caudal hindbrain (Figure 3E). This is consistent with the low neurogenic potential of the caudal hindbrain p3[5HT] domain. The block in generation of ectopic V3 neurons by comisexpression of *Nkx2.2-VP16* confirmed the derivation of these *Hes1*-induced ectopic V3 neurons from the presumptive p3[5HT] domain (Figure S3A). To further test the idea that Ascl1 expression level is important for p3[5HT] identity, we measured the expression of Ascl1 in *Ascl1* heterozygote mice, in which 5HT neuronal differentiation is intact [11]. No significant change in Ascl1 expression was detected (WT p3[5HT] expression: 151.1 [mean] ± 53.2 [SD]; *Ascl1*^*+/−*^ p3[5HT] expression: 149.3 ± 39.7), which implies that allele number is not the main determinant of the level of Ascl1 expression (Figure S3B). Together, these data support the case for the critical role of Ascl1 expression level in the selection of 5HT over V3 neuronal fate.

**Figure 4:**
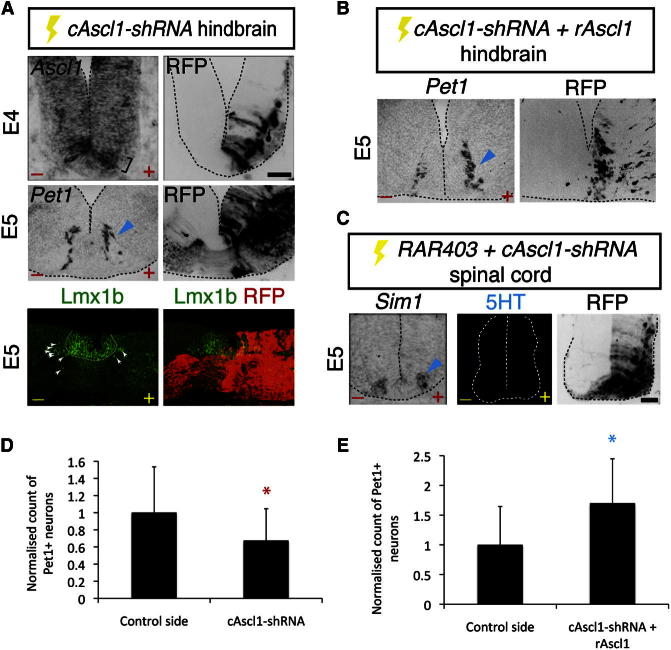
Knockdown of Chick *Ascl1* Alters p3 Progenitor Identity (A) Misexpression of *cAscl1-shRNA IRES RFP* decreases *Ascl1* expression in hindbrain p3 progenitors (bracket) and reduces *Pet1* (arrowhead) and Lmx1b expression (short arrows). Dotted white line demarcates Lmx1b expression corresponding to the floor plate. RFP expression on the control side is confined to decussating axons. Scale bar represents 18 μm. (B) Coelectroporation of *cAscl1-shRNA* and *rAscl1* rescues *Pet1* expression (arrowhead). (C) *cAscl1-shRNA* restores *Sim1*^*+*^ V3 neurons (arrowhead) and blocks ectopic spinal 5HT expression induced by *RAR403*. Scale bar represents 13 μm. (D and E) Quantification of *Pet1*^*+*^ neurons in the caudal hindbrain at E5 following forced expression of *cAscl1-shRNA* (D) or *cAscl1-shRNA* and *rAscl1* (E). Red asterisk, p = 0.028; blue asterisk, p = 0.002. See also Figure S3.

Finally, to test directly whether the level of Ascl1 determines p3 progenitor fate, we used *cAscl1-shRNA*. Forced expression of *cAscl1-shRNA* in the ventral region of the caudal hindbrain reduced the expression of *Ascl1* in hindbrain p3 progenitors (control p3[5HT] expression: 213.6 [mean] ± 22.8 [SD]; *cAscl1-shRNA* p3[5HT] expression: 123.9 ± 13.3; control p3[V3] expression: 119.5 ± 19.5), and this was accompanied by a ∼40% reduction in 5HT neuronal differentiation (Figures 4A and 4D). Consistent with the retinoid and Notch pathway gain-of-function experiments in the caudal hindbrain in ovo, occasional ectopic V3 neurons were also detected (Figure S3C). We then attempted to rescue these neural patterning defects by comisexpression of rat Ascl1 (rAscl1), which is predicted to be resistant to this shRNA (Figure 4B). *rAscl1* restored serotonergic neurogenesis and prevented the ectopic generation of any V3 neurons by *cAscl1-shRNA* (Figures 4B and 4E; Figure S3D). Transfection of a scrambled version of the chick *Ascl1* shRNA (*cAscl1-shRNA-SCR*) did not alter the expression of *Ascl1* in p3[5HT] progenitors (control p3[5HT] expression = 185.9 [mean] ± 30.1 [SD]; *cAscl1-shRNA-SCR* p3[5HT] expression = 176.0 ± 38.5), nor did it alter 5HT neurogenesis or induce any ectopic V3 neurons (Figures S3E and S3F). Furthermore, when the increased expression of *Ascl1* in p3[V3] progenitors elicited by *RAR403* was prevented by comisexpression of *cAscl1-shRNA* (Figure 2E), V3 neuronal differentiation was rescued (V3 neurons in control = 11 [mean] ± 4.6 [SD]; V3 neurons following *RAR403 + cAscl1-shRNA* misexpression = 10.8 ± 5.1; p = 0.92, not significant), and ectopic 5HT neuronal generation was blocked (Figure 4C). Taken together, these data indicate that Ascl1 recapitulates the effect of retinoids on p3 progenitor identity and that retinoids regulate the neuronal fates of p3 progenitors in an *Ascl1*-dependent manner.

## Discussion

We have shown how different profiles of retinoid signaling in p3 progenitors that share a common transcriptional code generate distinct neuronal identities at spinal cord and hindbrain levels. Binary differences in retinoid activity result in graded shifts in expression of a pivotal transcription factor, Ascl1, which is critical for the selection of alternate progenitor identities. This mechanism of progenitor fate specification is distinct from the conventional combinatorial model, because allocation to V3 or 5HT neuronal identity is determined not by qualitative but by quantitative differences in the transcriptional code [1]. Moreover, this model does not exclude the possibility that other, as yet uncharacterized, intrinsic determinants function in the same pathway as Ascl1 and could discriminate p3[5HT] from p3[V3].

In invertebrates, graded activity of transcription factors has been shown to control differential gene expression and thereby diversify cell fates [31]. By contrast in vertebrates, in general, transcription factor gradients appear to refine neural identity rather than instruct distinct cell identities in a dose-dependent manner [32–36]. Consistent with their closely similar genetic identities, the manipulations of retinoid signaling and Ascl1 expression demonstrate that spinal p3 progenitors possess a cryptic bipotency. The induction of ectopic V3 neurons in the hindbrain is less robust, which implies that hindbrain p3 progenitors are not fully interconvertible by graded Ascl1 expression alone. Nevertheless, it is clear that *Ascl1* expression must be tightly regulated to regionally constrain 5HT neurogenesis. Importantly, failure to appropriately regulate the number of 5HT neurons is associated with neuropsychiatric disease states, for example Smith-Lemli-Opitz syndrome, in which hypermorphic serotonergic differentiation occurs [37].

## Supplemental Information

Document S1. Figures S1–S3 and Supplemental Experimental Procedures
