# Retinoid Acid Specifies Neuronal Identity through Graded Expression of Ascl1

**DOI:** 10.1016/j.cub.2013.03.017

**Published:** 2013-04-08

**Authors:** John Jacob, Jennifer Kong, Steven Moore, Christopher Milton, Noriaki Sasai, Rosa Gonzalez-Quevedo, Javier Terriente, Itaru Imayoshi, Ryoichiro Kageyama, David G. Wilkinson, Bennett G. Novitch, James Briscoe

(Current Biology *23*, 412–418; March 4, 2013)

As a result of an author oversight at the proof stage, author Ryoichiro Kageyama’s first name was initially misspelled as “Ryoichoro” in this article online and in print. This error has now been corrected in the article online. The authors apologize for the error and any confusion that may have resulted.

