# Supplementary material for: Retinoid Acid Specifies Neuronal Identity through Graded Expression of Ascl1
Source: Curr Biol. 2013 Mar 4;23(5):412–8. doi: 10.1016/j.cub.2013.01.046 (PMC3659286; doi:10.1016/j.cub.2013.01.046)
Supplement: Document S1. Figures S1–S3 and Supplemental Experimental Procedures [file mmc1.pdf]

Current Biology, Volume *23*

Supplemental Information

Retinoid Acid Specifies Neuronal Identity through Graded Expression of Ascl1

John Jacob, Jennifer Kong, Steven Moore, Christopher Milton, Noriaki Sasai, Rosa Gonzalez-Quevedo, Javier Terriente, Itaru Imayoshi, Ryoichoro Kageyama, David G. Wilkinson, Bennett G. Novitch, and James Briscoe

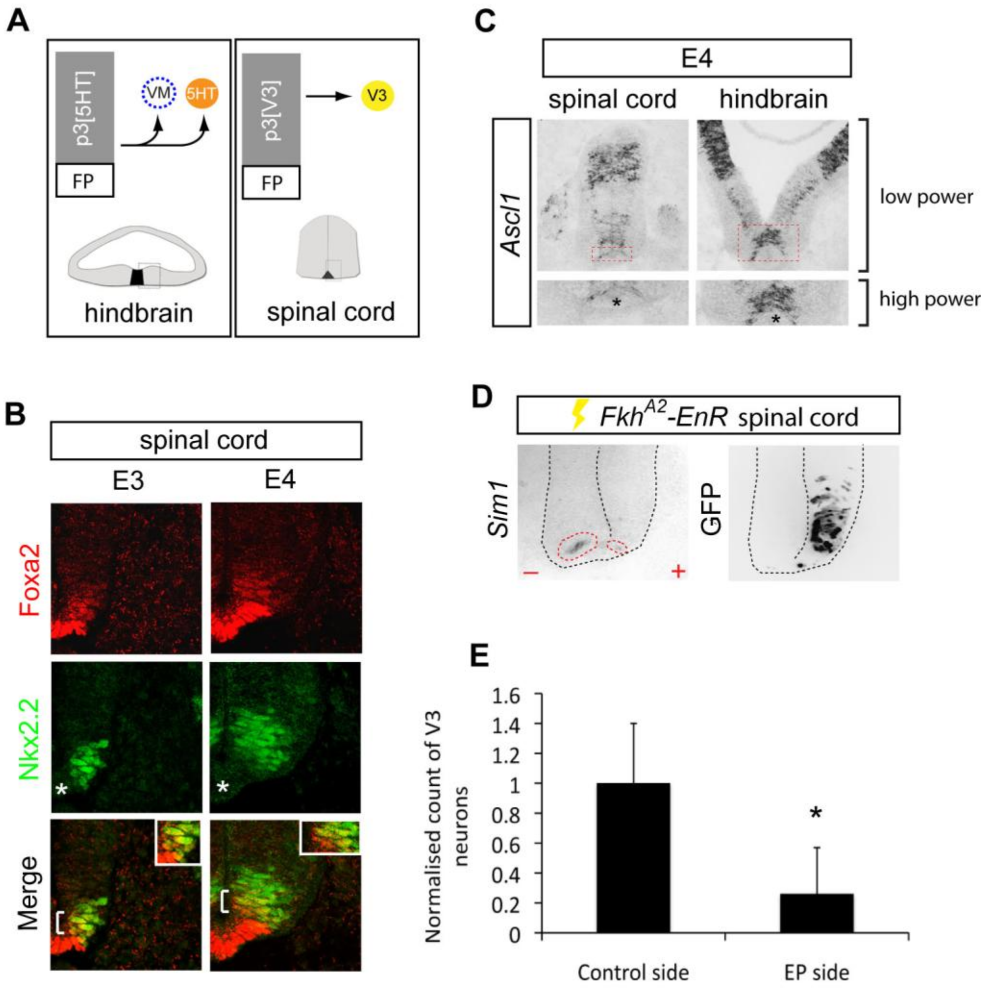

Figure S1, Related to Figure 2. Fate Determinants that Specify the p3 Domain of the Spinal Cord

(A) Schematic showing the different neuronal progeny derived from p3 progenitors in the hindbrain and spinal cord. VM, visceral motor.

(B) Horizontal sections of the rostral spinal cord of the chick showing co-expression of Foxa2 (red) and Nkx2.2 (green) by immunofluorescence. The region of overlap is indicated by a square bracket, and is shown at high power in the insets.

(C) *Ascl1* expression detected by in-situ hybridisation in the p3 domain (boxed) of the rostral spinal cord (left) and caudal hindbrain (right) of the chick. Lower panels show high power views of *Ascl1* expression in the boxed regions. In (B and C) the asterisk marks the floor plate.

(D) Forced expression of a dominant negative version of Foxa2, *Fkh*^{A2}*-EnR IRES GFP* in chick p3[V3] progenitors markedly reduces the expression of *Sim1*, a marker of V3 neurons.

(E) Quantification of *Sim1*$^{+}$ V3 neurons at E5 following in ovo electroporation of *Fkh*^{A2}*-EnR IRES GFP*. * $p = 2.05 \times 10^{-11}$. Error bars represent SD.

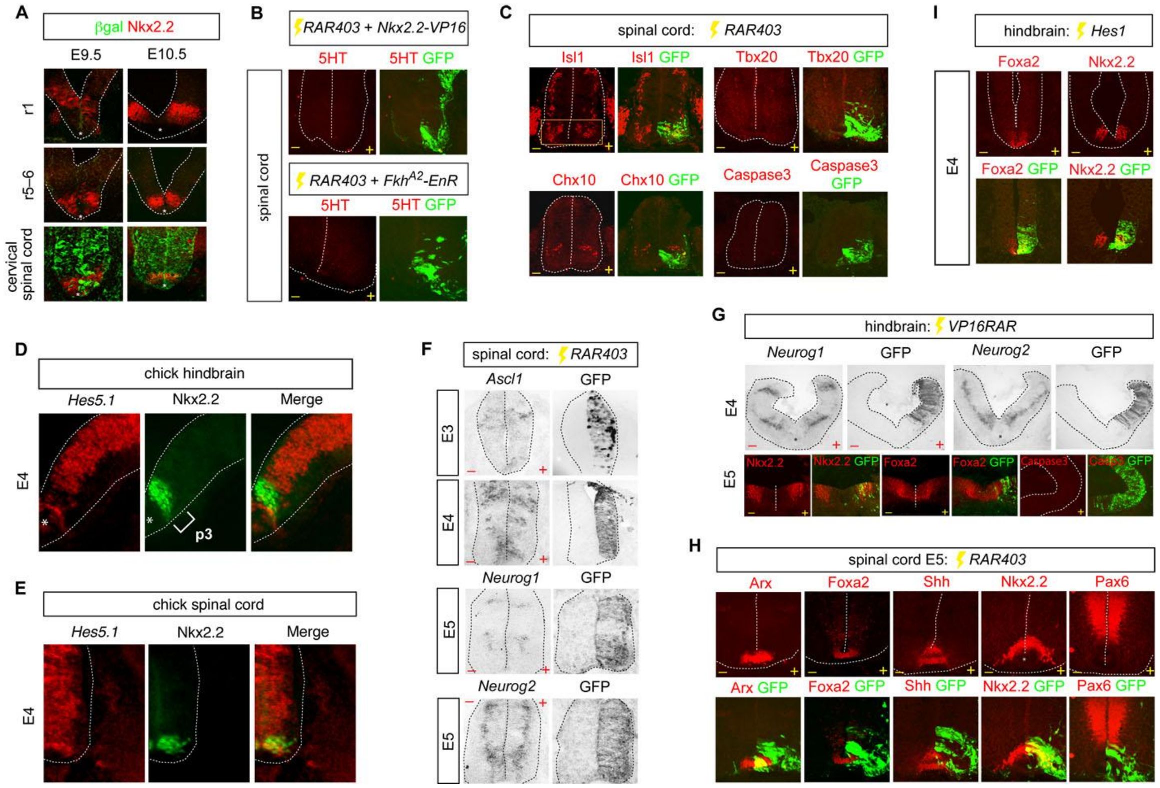

Figure S2, Related to Figure 1. Effect of Retinoid Signaling on Ventral Neural Progenitor Identities in Developing Mouse and Chick Embryos

(A) In a transgenic mouse line harbouring a retinoic acid response element fused to the beta-galactosidase gene (*RARE-LacZ*), βgal (green) detection by immunofluorescence reports the spatial distribution of retinoid signalling. The p3 domain in the hindbrain and spinal cord is marked by immunostaining for Nkx2.2 (red). Representative sections are shown at the indicated axial levels. r, rhombomere.

(B) Ectopic spinal cord 5HT neurons are derived from p3 progenitors. In ovo electroporation of *RAR403* and either a dominant negative version of Nkx2.2, *Nkx2.2-VP16* (upper panels) or Foxa2, *Fkh^{A2}-EnR* (lower panels) prevents ectopic 5HT neuronal generation in the brachial level spinal cord as shown by the absence of 5HT immunostaining.

(C) Blockade of retinoid signalling in the chick by *RAR403* misexpression in p3[V3] progenitors does not affect patterning of other neuronal subtypes, the Isl1^{+} somatic motor neurons and Chx10^{+} V2 interneurons in the ventral spinal cord. Moreover, ectopic VM neurons, marked by Tbx20 expression are not induced. *RAR403* misexpression does not result in increased cell death in the ventral spinal cord as indicated by the absence of activated Caspase3 immunostaining.

(D and E**)** Expression of the Notch pathway gene, *Hes5.1* in Nkx2.2^{+} p3[5HT] (D), and p3[V3] (E), progenitors.

(F) In-situ hybridisation for *Ascl1* at E3 and E4 and the classic proneural genes, *Neurog1* and *Neurog2* at E5, following *RAR403 IRES GFP* misexpression in the spinal cord.

(G) Following in ovo *VP16RAR* misexpression in the caudal hindbrain no change in *Neurog1* or *Neurog2* expression is detected. Expression of the p3[5HT] progenitor markers, Nkx2.2 and Foxa2 is not altered by *VP16RAR* misexpression. Assay for cell death by immunostaining for

activated Caspase3 shows that *VP16RAR* misexpression does not induce cell death in the hindbrain.

(H) Immunofluorescence images of the floor plate markers, Arx and sonic hedgehog (Shh) and the ventral progenitor markers Foxa2, Nkx2.2 and Pax6 following *RAR403* misexpression in the p3[V3] domain.

(I) Expression of progenitor markers FoxA2 and Nkx2.2 in the caudal hindbrain at E4 following forced expression of *Hes1-IRES GFP*.

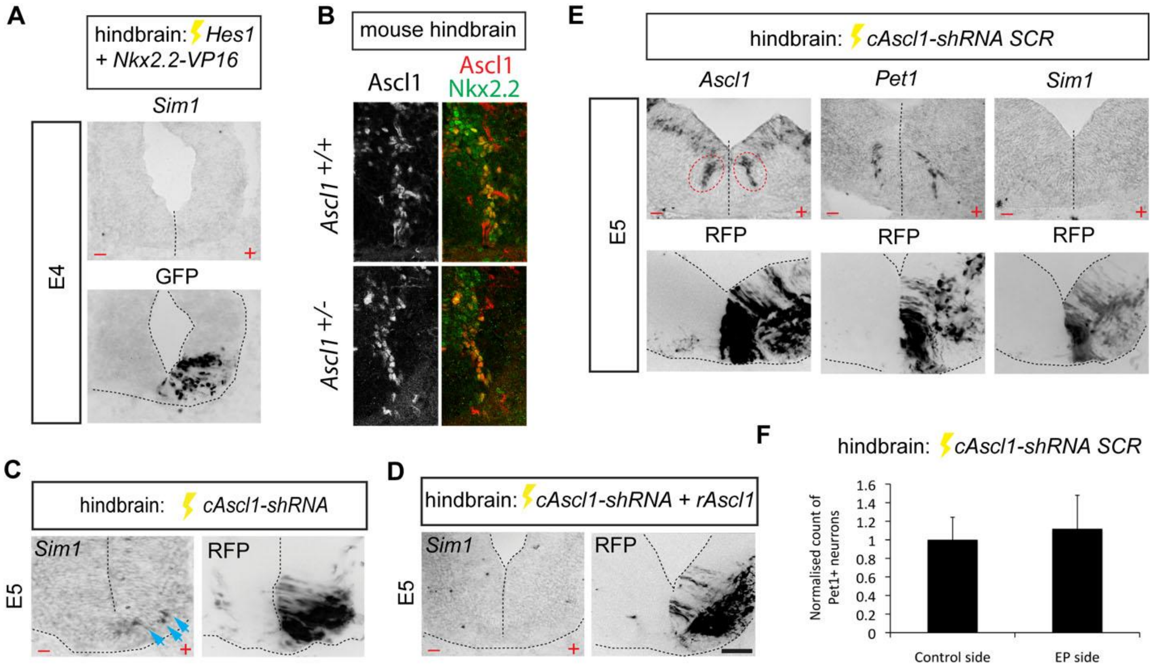

Figure S3, Related to Figure 4. Effect on Neural Patterning of Lowering *Ascl1* Expression in p3[5HT] Progenitors in the Chick, by *Hes1* In Ovo Electroporation, or Directly by *Ascl1* Knockdown In Ovo

(A) Absence of ectopic expression of *Sim1*, the V3 neuronal marker in the caudal hindbrain at E4 following co-electroporation of *Hes1* and *Nkx2.2-VP16*.

(B) Horizontal sections through the caudal hindbrain of E11.5 WT ($Ascl1^{+/+}$) and Ascl1 heterozygous ($Ascl1^{+/-}$) mouse embryos showing Ascl1 expression in hindbrain p3 progenitors (marked by Nkx2.2 expression in green). Embryos were imaged using identical settings in the same confocal session.

(C) Misexpression of *cAscl1-shRNA IRES RFP* induces occasional ectopic $Sim1^{+}$ V3 neurons (arrows) in the ventral region of the caudal hindbrain.

(D) Following co-misexpression of *cAscl1-shRNA* and *rAscl1* in ovo, ectopic V3 neurons are never detected in the caudal hindbrain.

(E) Lack of effect of control scrambled *cAscl1-shRNA* (*cAscl1-shRNA SCR*) on *Ascl1,* 5HT neuronal differentiation, marked by *Pet1* expression, and V3 differentiation, marked by *Sim1*, at E5 in the caudal hindbrain of chick. *Ascl1* expression corresponding to the p3[5HT] domain is circled.

(F) Quantification of 5HT neuronal differentiation, using *Pet1* as a marker, in the caudal hindbrain of the chick following forced expression of *cAscl1-shRNA SCR*. p = 0.38, N.S.

Supplemental Experimental Procedures

Immunohistochemistry and In-Situ Hybridisation

Localisation of proteins and mRNA was performed using antibodies or riboprobes as described [1-3]. Rabbit polyclonal antibodies were used to detect β-galactosidase (Biogenesis, Poole UK). The Ascl1 expression level was assayed in p3 progenitors cells by determining the mean pixel intensity (ImageJ) within a traced outline of individual nuclei on one or both sides of the neural tube at the developmental stages and axial levels described. For each measurement the mean background intensity was initially determined by measuring the mean pixel intensity in an area of tissue not expressing Ascl1, away from the p3 domain. The mean background intensity per pixel was calculated by dividing the mean intensity in the defined area by the number of pixels in that area. The mean background intensity/pixel was multiplied by the number of pixels in the region of interest (p3 domain progenitor) to derive the mean background intensity for each p3 progenitor. This value was subtracted from the mean intensity of Ascl1 expression in the region of interest to obtain the background corrected mean intensity per cell. These calculations were iterated across the population of p3 progenitor cells in each experimental condition to obtain an overall mean intensity. Cells that failed to express GFP/RFP on the electroporated side were excluded from the analysis. For any given experiment, all sections were processed under the same conditions and imaged during the same session using identical microscope settings. The statistical significance of the differences in neuronal counts in the various experimental conditions was tested using the unpaired Student's ttest. In all graphs the data range is represented by the standard deviation (SD).

Expression Constructs and Mouse Lines

zCyp26B1 cDNA was cloned into the EcoRV and Sal1 restriction sites of a *miniTol2 HSP-UAS-α-crystallin RFP* plasmid in frame with 3' *myc* tag sequences using the In-Fusion PCR Cloning System (Clontech). To drive the expression of *zCyp26B1* in ovo, a *pCS2* vector containing *GAL4* (KalTA4) [4] was coelectroporated together with a *UAS-GFP* reporter plasmid (gift from Dr Q. Xu). To construct the *Hes1* expression vector, chicken *Hes1* was amplified by RT-PCR and an approximately 900 bp fragment was subcloned into the *pCAGGS* vector at Nhe1/Xho1 restriction sites upstream of *IRES GFP* using the following pair of primers: 5'-tatagctagcatgcccgccgacacgggcatggaaaaaccc-3' and 5'-tatactcgagtcaccacggcctccagacggactccctgcg-3'. Expression vectors for *Fkh*^{A2}*-EnR* [1], *Nkx2.2-VP16* [5], *RAR403* [6], *VP16RAR* [7], *Ascl1* and *Neurog2* [8], *Foxa2* [1] and *Nkx2.2* [5] have been described. *cAscl1-shRNA* was constructed using the following primer pairs: 5'-gagaggtgctgctgagcgagagtcaagctggtgaacctttagtgaagccacagatgta-3'; 5'-attcaccaccactaggcacgagtcaagctggtgaacctttacatctgtggcttcact-3' [9]. *cAscl1-shRNA SCR* was constructed using the primer pair: 5'-gagaggtgctgctgagcgatcgaacgtcgtcggacttaatagtgaagccacagatgta-3' and 5'-attcaccaccactaggcagtcgaacgtcgtcggacttaatacatctgtggcttcact-3'. In ovo electroporation was performed at Hamburger-Hamilton stages 10-12 [10]. For each condition, observations were based on analysis of at least 4 or 5 embryos. Cell counts were derived from at least 2 sections/embryo from a minimum of 3 embryos. The *RARE-LacZ* [11], *Rbpj*$^{flox/+}$ [12], *Hes5* [13], *Ascl1* [14] and *Olig2-Cre/+*, *ROSA26* [15] mouse lines were generated as described.

Supplemental References

1. Jacob, J., Ferri, A.L., Milton, C., Prin, F., Pla, P., Lin, W., Gavalas, A., Ang, S.L., and Briscoe, J. (2007). Transcriptional repression coordinates the temporal switch from motor to serotonergic neurogenesis. Nature Neuroscience *10*, 1433-1439.
2. Jacob, J., Storm, R., Castro, D.S., Milton, C., Pla, P., Guillemot, F., Birchmeier, C., and Briscoe, J. (2009). Insm1 (IA-1) is an essential component of the regulatory network that specifies monoaminergic neuronal phenotypes in the vertebrate hindbrain. Development (Cambridge, England) *136*, 2477-2485.
3. Perez, S.E., Rebelo, S., and Anderson, D.J. (1999). Early specification of sensory neuron fate revealed by expression and function of neurogenins in the chick embryo. Development (Cambridge, England) *126*, 1715-1728.
4. Distel, M., Wullimann, M.F., and Koster, R.W. (2009). Optimized Gal4 genetics for permanent gene expression mapping in zebrafish. Proc Natl Acad Sci U S A *106*, 13365-13370.
5. Muhr, J., Andersson, E., Persson, M., Jessell, T.M., and Ericson, J. (2001). Groucho-mediated transcriptional repression establishes progenitor cell pattern and neuronal fate in the ventral neural tube. Cell *104*, 861-873.
6. Damm, K., Heyman, R.A., Umesono, K., and Evans, R.M. (1993). Functional inhibition of retinoic acid response by dominant negative retinoic acid receptor mutants. Proc Natl Acad Sci U S A *90*, 2989-2993.
7. Castro, D.S., Arvidsson, M., Bondesson Bolin, M., and Perlmann, T. (1999). Activity of the Nurr1 carboxyl-terminal domain depends on cell type and integrity of the activation function 2. The Journal of biological chemistry *274*, 37483-37490.
8. Castro, D.S., Skowronska-Krawczyk, D., Armant, O., Donaldson, I.J., Parras, C., Hunt, C., Critchley, J.A., Nguyen, L., Gossler, A., Gottgens, B., et al. (2006). Proneural bHLH and Brn proteins coregulate a neurogenic program through cooperative binding to a conserved DNA motif. Developmental cell *11*, 831-844.
9. Das, R.M., Van Hateren, N.J., Howell, G.R., Farrell, E.R., Bangs, F.K., Porteous, V.C., Manning, E.M., McGrew, M.J., Ohyama, K., Sacco, M.A., et al. (2006). A robust system for RNA interference in the chicken using a modified microRNA operon. Developmental biology *294*, 554-563.
10. Stamataki, D., Ulloa, F., Tsoni, S.V., Mynett, A., and Briscoe, J. (2005). A gradient of Gli activity mediates graded Sonic Hedgehog signaling in the neural tube. Genes & development *19*, 626-641.
11. Rossant, J., Zirngibl, R., Cado, D., Shago, M., and Giguere, V. (1991). Expression of a retinoic acid response element-hsplacZ transgene defines specific domains of transcriptional activity during mouse embryogenesis. Genes & development *5*, 1333-1344.
12. Han, H., Tanigaki, K., Yamamoto, N., Kuroda, K., Yoshimoto, M., Nakahata, T., Ikuta, K., and Honjo, T. (2002). Inducible gene knockout of transcription factor recombination signal binding protein-J reveals its essential role in T versus B lineage decision. International immunology *14*, 637-645.
13. Hatakeyama, J., Bessho, Y., Katoh, K., Ookawara, S., Fujioka, M., Guillemot, F., and Kageyama, R. (2004). Hes genes regulate size, shape and histogenesis of the nervous system by control of the timing of neural stem cell differentiation. Development (Cambridge, England) *131*, 5539-5550.

14. Guillemot, F., Lo, L.C., Johnson, J.E., Auerbach, A., Anderson, D.J., and Joyner, A.L. (1993). Mammalian achaete-scute homolog 1 is required for the early development of olfactory and autonomic neurons. Cell *75*, 463-476.
15. Dessaud, E., Yang, L.L., Hill, K., Cox, B., Ulloa, F., Ribeiro, A., Mynett, A., Novitch, B.G., and Briscoe, J. (2007). Interpretation of the sonic hedgehog morphogen gradient by a temporal adaptation mechanism. Nature *450*, 717-720.
